# Activation of Transient Receptor Potential Vanilloid 4 Impairs the Dendritic Arborization of Newborn Neurons in the Hippocampal Dentate Gyrus through the AMPK and Akt Signaling Pathways

**DOI:** 10.3389/fnmol.2017.00190

**Published:** 2017-06-15

**Authors:** Yujing Tian, Mengwen Qi, Zhouqing Wang, Chunfeng Wu, Zhen Sun, Yingchun Li, Sha Sha, Yimei Du, Lei Chen, Ling Chen

**Affiliations:** ^1^Department of Physiology, Nanjing Medical UniversityNanjing, China; ^2^Department of Neurology, Children’s Hospital of Nanjing Medical UniversityNanjing, China; ^3^Department of Tangshan Branch, Jinling Hospital, Nanjing UniversityNanjing, China; ^4^Research Center of Ion Channelopathy, Institute of Cardiology, Union Hospital, Tongji Medical College, Huazhong University of Science and TechnologyWuhan, China; ^5^Neuroprotective Drug Discovery Center, Nanjing Medical UniversityNanjing, China

**Keywords:** TRPV4, dendritic arborization, calcium, AMPK, Akt

## Abstract

Neurite growth is an important process for the adult hippocampal neurogenesis which is regulated by a specific range of the intracellular free Ca^2+^ concentration ([Ca^2+^]_i_). Transient receptor potential vanilloid 4 (TRPV4) is a calcium-permeable channel and activation of it causes an increase in [Ca^2+^]_i_. We recently reported that TRPV4 activation promotes the proliferation of stem cells in the adult hippocampal dentate gyrus (DG). The present study aimed to examine the effect of TRPV4 activation on the dendrite morphology of newborn neurons in the adult hippocampal DG. Here, we report that intracerebroventricular injection of the TRPV4 agonist GSK1016790A for 5 days (GSK1016790A-injected mice) reduced the number of doublecortin immunopositive (DCX^+^) cells and DCX^+^ fibers in the hippocampal DG, showing the impaired dendritic arborization of newborn neurons. The phosphorylated AMP-activated protein kinase (p-AMPK) protein level increased from 30 min to 2 h, and then decreased from 1 to 5 days after GSK1016790A injection. The phosphorylated protein kinase B (p-Akt) protein level decreased from 30 min to 5 days after GSK1016790A injection; this decrease was markedly attenuated by the AMPK antagonist compound C (CC), but not by the AMPK agonist AICAR. Moreover, the phosphorylated mammalian target of rapamycin (mTOR) and p70 ribosomal S6 kinase (p70S6k) protein levels were decreased by GSK1016790A; these changes were sensitive to 740 Y-P and CC. The phosphorylation of glycogen synthase kinase 3β (GSK3β) at Y^216^ was increased by GSK1016790A, and this change was accompanied by increased phosphorylation of microtubule-associated protein 2 (MAP2) and collapsin response mediator protein-2 (CRMP-2). These changes were markedly blocked by 740 Y-P and CC. Finally, GSK1016790A-induced decrease of DCX^+^ cells and DCX^+^ fibers was markedly attenuated by 740 Y-P and CC, but was unaffected by AICAR. We conclude that TRPV4 activation impairs the dendritic arborization of newborn neurons through increasing AMPK and inhibiting Akt to inhibit the mTOR-p70S6k pathway, activate GSK3β and thereby result in the inhibition of MAP2 and CRMP-2 function.

## Introduction

It has been proven that the mammalian brain continuously produces newborn neurons in the hippocampal dentate gyrus (DG) throughout adulthood (Alvarez-Buylla and Garcia-Verdugo, 2002). Adult hippocampal neurogenesis is a complex process that begins with stem cells proliferation and followed by neuronal lineage specification, maturation, migration and incorporation into the hippocampal circuitry (Ge et al., 2008). Deficits in adult hippocampal neurogenesis can be seen in the pathogenesis of neurological diseases, including Alzheimer’s disease (AD), Parkinson’s disease (PD), depression and epilepsy (Balu and Lucki, 2009). Neurite growth is an important process in adult hippocampal neurogenesis and this process is crucial for the newborn neurons to establish new synaptic connections with the existing hippocampal circuitry by extending axonal and dendritic projections (Zhao et al., 2006). Neurite growth is regulated by a specific range of the intracellular free calcium concentration ([Ca^2+^]_i_) (Mattson, 1987; Zheng, 2007; Toth et al., 2016). Sustained elevation of [Ca^2+^]_i_ inhibits growth cone advancement, and maximal neurite outgrowth occurred within an optimal range of Ca^2+^ concentrations (Tang and Dent, 2003). Ca^2+^ influx through Ca^2+^-permeable ion channels on the cell membrane or release from the intracellular Ca^2+^ stores can increase [Ca^2+^]_i_. In neurons, Ca^2+^ influx mainly occurs through voltage-gated calcium channels (VGCCs) and ligand-gated ion channels such as *N*-methyl-D-aspartate glutamate receptor (NMDAR), nicotinic acetylcholine receptor (nAChR), and transient receptor potential (TRP) channels (Pankratov and Lalo, 2014; Benemei et al., 2015). It has been reported that an L-type VGCC antagonist promotes axon outgrowth in dopaminergic brain slice co-cultures (Sygnecka et al., 2015). NMDAR has been proven to regulate neurite growth in the hippocampus (Nacher and McEwen, 2006). Activation of α7nAChR significantly reduces axon growth in hippocampal neurons and thereby contributes to synaptic development and plasticity in the hippocampus (Nordman et al., 2014). Therefore, Ca^2+^-permeable channels may provide effective targets for the regulation of neurite growth.

The TRP channel family consists of several non-selective cationic channels that are sensitive to multiple physicochemical stimuli and capable of coupling their activity to downstream modulations of intracellular signals (Benemei et al., 2015). TRP vanilloid 4 (TRPV4) is a member of the vanilloid TRP subfamily (White et al., 2016). Activation of TRPV4 induces Ca^2+^ influx, which increases [Ca^2+^]_i_ in various cell types. It has been reported that TRPV4 mediates neurotrophic factor-derived neuritogenesis in developing peripheral neurons (Jang et al., 2012). Some mutations in *TRPV4* that augment Ca^2+^ influx have been shown to underlie the pathogenesis of TRPV4-linked axonal neuropathies (Fecto et al., 2011). In our recent study, activation of TRPV4 promoted the proliferation of stem cells in the adult hippocampal DG (Tian et al., 2016). Whether activation of TRPV4 participates in the modulation of the neurite growth of newborn neurons in the hippocampal DG remains unclear. Activation of AMP-activated protein kinase (AMPK) has been shown to regulate the hippocampal neuronal structure (Ramamurthy et al., 2014). In addition to playing a pivotal role in modulating neuronal survival and differentiation, protein kinase B (Akt) has recently emerged as an important regulator of neurite outgrowth (Read and Gorman, 2009). Activation of TRPV4 has been reported to modulate the AMPK and Akt signaling pathways *in vitro* (Hong et al., 2016). In the present study, we sought to determine whether TRPV4 activation affects the dendrite morphology of newborn neurons and further explored whether the AMPK or Akt signaling pathway is involved in TRPV4 action.

## Materials and Methods

### Experimental Animals

Male mice (9–10 weeks old; ICR, Oriental Bio Service Inc., Nanjing, China) were used in the experiments. This study was approved by Animal Care and Ethical Committee of Nanjing Medical University. All animal experiments were conducted in accordance with the Guide for the Care and Use of Laboratory Animals of Nanjing Medical University. Animals were housed under standard conditions (room temperature 23 ± 2°C, humidity 55 ± 5% and 12:12 h light/dark cycle) in the Animal Care Facility of Nanjing Medical University and were permitted free access to food and water. All efforts were made to minimize the animals’ suffering and to reduce the number of animals used.

### Drug Treatment

GSK1016790A is a synthetic agonist of TRPV4 and has been used to activate TRPV4 both *in vivo* and *in vitro* (Vincent and Duncton, 2011; Jie et al., 2015, 2016; Hong et al., 2016). AICAR is a commonly used and specific activator of AMPK, and compound C (CC) is a specific antagonist of AMPK (Han et al., 2005; Venna et al., 2012). 740 Y-P (or PDGFR ^740^Y-P) can bind with high affinity to p85 subunit of PI3K and has been widely used as a special agonist of PI3K (Derossi et al., 1998; Williams and Doherty, 1999). GSK1016790A, AICAR and 740 Y-P were administered by intracerebroventricular (icv) injections as described in previous reports (Han et al., 2005; Jie et al., 2015, 2016). Briefly, these drugs were injected into the right lateral ventricle (0.3 mm posterior, 1.0 mm lateral, and 2.5 mm ventral to the bregma) using a stepper motor-controlled microsyringe (Stoelting, Wood Dale, IL, United States) at a rate of 0.2 μl/min. The lateral ventricle within each cerebral hemisphere may communicate with the third ventricle through an interventricular foramen. To avoid the possible effect of these drugs on the left hippocampus, control mice were injected with the same volume of vehicle into the right lateral ventricle. It has been reported that CC showed neuronal protection in experimental stroke rodent models when it was administered intraperitoneally (ip) (Li J. et al., 2010; Liu et al., 2012; Venna et al., 2012). Therefore, in this study, CC was ip injected as previously reported (Li J. et al., 2010; Liu et al., 2012; Venna et al., 2012). GSK1016790A and CC were first dissolved in DMSO and then in 0.9% saline with a DMSO concentration of 1%. GSK1016790A (0.1 to 5 μM/mouse) was injected once daily for 5 days (GSK1016790A-injected mice). CC (10 mg/Kg), AICAR (2 mM/mouse) or 740 Y-P (30 μM/mouse) was injected 30 min before GSK1016790A injection and then injected once daily for 5 days. The concentrations of these drugs were chosen based on previous reports (Han et al., 2005; Li J. et al., 2010; Liu et al., 2012; Venna et al., 2012; Jie et al., 2015, 2016). The final volume was 2 μl for icv and 0.2 ml for ip injection. Control mice were injected with the same volume of vehicle. Each experimental group contained 9 mice.

### Histological Examination

The mice were anesthetized with chloral hydrate (400 mg/kg, ip) and transcardially perfused with 4% paraformaldehyde 5 days after the beginning of the experiment (i.e., the first GSK1016790A injection). Brains were post-fixed overnight in 4% paraformaldehyde at 4°C and coronally sectioned (40 μM thick) using a vibrating microtome (Microslicer DTK 1500; Dousaka EM Co., Kyoto, Japan). For doublecortin (DCX) staining, free-floating sections were incubated with goat polyclonal anti-DCX antibody (1:500; Santa Cruz Biotechnology; Santa Cruz, CA, United States) overnight at 4°C; they were then incubated with biotin-labeled rabbit anti-goat IgG antibody (1:200; Bioworld Technology, St. Louis Park, MN, United States) for 2 h at room temperature.

The DCX immunopositive (DCX^+^) cells in the sub-granular zone (SGZ) and DCX^+^ fibers in the molecular layer (ML) of the hippocampal DG were counted using a conventional light microscope (Olympus DP70, Tokyo, Japan) with an oil immersion lens (100×) and analyzed with the ImageJ software (NIH Image, Bethesda, MD, United States) (Wang et al., 2015). The DCX^+^ cells were expressed as the number of DCX^+^ cells per mm length along SGZ (Wang et al., 2015). The number of DCX^+^ fibers within 1 mm length was divided by the number of DCX^+^ cells. The number of DCX^+^ fibers in the middle third of the ML (Mm) stands for the density of dendrite and that in the outer third of the ML (Mo) stands for the length of dendrite (Sha et al., 2013; Wang et al., 2015).

### Western Blot Analysis

After the mice were decapitated under deep anesthesia with ethyl ether, the hippocampi were quickly removed. The hippocampal protein concentrations were determined using a BCA Protein Assay Kit (Pierce, Rochford, IL, United States). Equal levels of protein were separated by SDS-polyacrylamide gel electrophoresis and transferred to PVDF membranes. The membranes were blocked using 5% non-fat milk in Tris-buffered saline (TBS)/Tween-20 and then incubated with antibodies against phospho-AMPK (p-AMPK, 1:1000, Cat# 2531, Cell Signaling Technology, Beverly, MA, United States), phospho-Akt (p-Akt, 1:1000, Cat# 4060, Cell Signaling Technology, Beverly, MA, United States), phospho-mammalian target of rapamycin (p-mTOR, 1:1000, Cat# ab109268, Abcam, Cambridge, United Kingdom), phospho-p70 ribosomal S6 kinase (p-p70S6k, 1:1000, Cat# 9205, Cell Signaling Technology, Beverly, MA, United States), phospho-glycogen synthase kinase 3β (p-GSK3β, 1:1000, Cat# 612313, BD Bioscience, San Jose, CA, United States), phospho-microtubule-associated protein 2 (p-MAP2, 1:1000, Cat# 4544, Cell Signaling Technology, Beverly, MA, United States), phospho-collapsin response mediator protein-2 (p-CRMP-2, 1:1000, Cat# ab129066, Abcam, Cambridge, United Kingdom) and glyceraldehyde-3-phosphate dehydrogenase (GAPDH, 1:5000, Cat# ab181602, Abcam, Cambridge, United Kingdom) at 4°C overnight. After being washed with TBST, the membranes were incubated with HRP-labeled secondary antibody, developed using an ECL Detection Kit (Amersham Biosciences, Piscataway, NJ, United States) and analyzed using Image J software (NIH). Following visualization of p-AMPK, p-Akt, p-mTOR, p-p70S6k, p-GSK3β, p-MAP2, and p-CRMP-2, the blots were stripped by incubation in stripping buffer (Restore, Pierce Chemical Co, Rockford, IL, United States) for 5 min, re-blocked for 60 min with skim milk at room temperature, and then incubated with AMPK (1:1000, Cat# 2532, Cell Signaling Technology, Beverly, MA, United States), Akt (1:1000, Cat# 9272, Cell Signaling Technology, Beverly, MA, United States), mTOR, (Cat# 2972, Cell Signaling Technology, Beverly, MA, United States), p70S6k (1:1000, Cat# 2708, Cell Signaling Technology, Beverly, MA, United States), GSK3β (1:1000, Cat# ab32391, Abcam, Cambridge, United Kingdom), MAP2 (1:1000, Cat# 8707, Cell Signaling Technology, Beverly, MA, United States) and CRMP-2 (1:1000, Cat# ab129082, Abcam, Cambridge, United Kingdom) antibodies, respectively. Hippocampal samples collected from the hemispheres of three mice were considered a set for western blot analysis. The data represent the average of three experimental sets. Western blot analysis for the p-AMPK, AMPK, p-Akt and Akt protein levels was performed at 30 min, 2 h, 1 and 5 days after the beginning of the experiment, and the analysis of the p-mTOR, mTOR, p-p70S6k, p70S6k, p-GSK3β, GSK3β, p-MAP2, MAP2, p-CRMP-2, and CRMP-2 protein levels was performed 5 days after the beginning of the experiment.

### Chemicals

All chemicals, unless otherwise stated, were obtained from Sigma-Aldrich Co.

### Data Analysis

Data are expressed as the mean ± S.E.M, and statistical analyses were performed using SPSS software, version 16.0 (SPSS Inc., United States). ANOVA followed by Bonferroni’s *post hoc* test was used to evaluate the statistical significance, and the significance level was set at *p* < 0.05 and *p* < 0.01. The protein levels of p-AMPK, p-Akt, p-mTOR, p-p70S6k, p-GSK3β, p-MAP2, and p-CRMP-2 were first normalized to the protein level of AMPK, Akt, mTOR, p70S6k, GSK3β, MAP2, and CRMP-2, respectively. The protein levels in mice injected with GSK1016790A and/or the kinase antagonist (agonist) were expressed as the percentage of that in vehicle-injected mice (control mice). The decrease in the number of DCX^+^ fibers in Mm resulting from different doses of GSK1016790A was first normalized to the decrease caused by 10 μM/mouse GSK1016790A. The dose-response curve was fitted by the Hill equation, *a* = *a*_max_/[1+(EC_50_/C)*^n^*], where *n* is the Hill coefficient and the EC_50_ value is the dose of GSK1016790A that produced a 50% effect.

## Results

### Effect of the TRPV4 Activation on the Dendritic Arborization of Newborn Neurons in the Hippocampal DG

**FIGURE 1:**
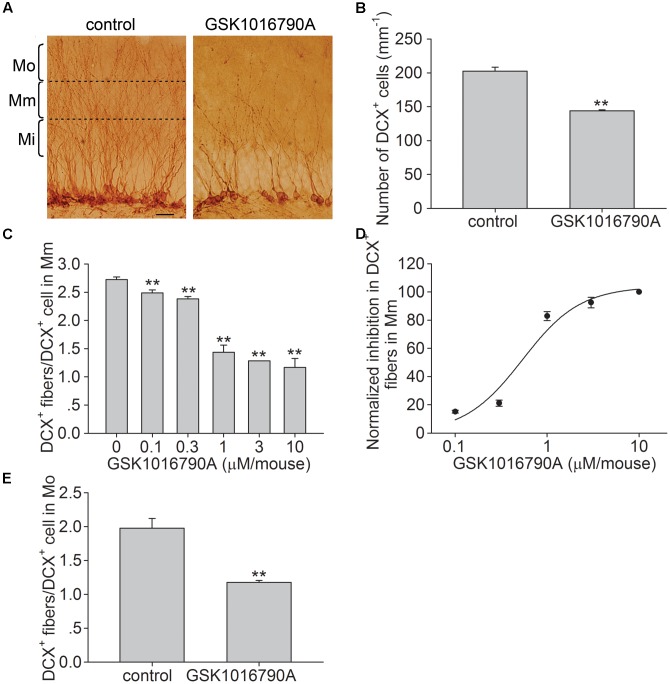
Effect of the TRPV4 activation on the dendritic arborization of newborn cells in the hippocampal DG. **(A)** Representative pictures of the DCX immunostaining in the hippocampal DG in the control and GSK1016790A-injected mice (GSK1016790A: 1 μM/mouse). The molecular layer of the DG was divided into the inner (Mi), middle (Mm), and outer (Mo) subregions. Scale bar = 25 μm. **(B)** The bar graph shows that the number of DCX^+^ cells was reduced by GSK1016790A treatment. **(C)** The bar graph shows DCX^+^ fibers in the Mm subregion of the DG decreased in the presence of different doses of GSK1016790A. **(D)** The dose-dependence curve for the GSK1016790A-induced decrease in DCX^+^ fibers in the Mm subregion. **(E)** The bar graph shows that the number DCX^+^ fibers in the Mo subregion was decreased by GSK1016790A (1 μM/mouse). ^∗∗^*p* < 0.01 vs. control mice.

Doublecortin, a microtubule-associated protein, is specifically expressed in newly generated neurons, with peak expression during the 2nd week after their birth (Rao and Shetty, 2004; Duan et al., 2008; Wang et al., 2015). In the present study, the administration of the TRPV4 agonist GSK1016790A for 5 days significantly decreased the number of DCX^+^ cells in the SGZ (**Figures 1A,B**), indicating that activation of TRPV4 may impair the newly generated neurons. DCX^+^ cells extend their dendrites in the direction of the ML (Rao and Shetty, 2004). The ML of the DG was divided into the inner (Mi), middle (Mm) and outer (Mo) subregions as previously reported (Wang et al., 2015; **Figure 1A**). The number of DCX^+^ fibers in the Mm subregion was lower in GSK1016790A-injected mice than in the control group (*p* < 0.01). At doses ranging from 0.1 to 10 μM/mouse, the number of DCX^+^ fibers in the Mm subregion was dose-dependently decreased by GSK1016790A, and the values of the Hill coefficient and the EC_50_ were 1.37 and 0.54 ± 0.14 μM/mouse, respectively (**Figures 1A,C,D**). After the administration of 1 μM/mouse GSK1016790A, the number of DCX^+^ fibers in the Mm subregion decreased by 47.69 ± 2.70% (*p* < 0.01), and this dose was used in the subsequent experiments. Compared to the control value, the number of DCX^+^ fibers in the Mo subregion was also significantly reduced in GSK1016790A-injected mice (*p* < 0.01). These results indicate that activation of TRPV4 impairs the dendritic arborization of newborn neurons.

### Effect of TRPV4 Activation on the AMPK and Akt Signaling Pathways

**FIGURE 2:**
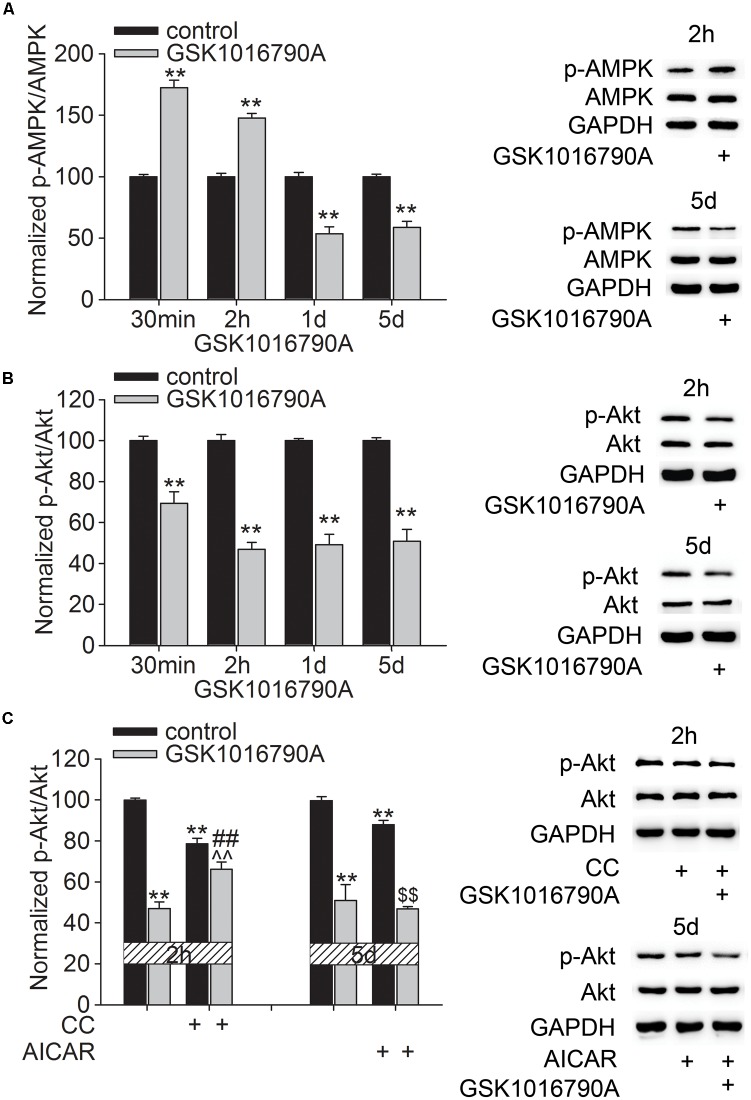
Effect of TRPV4 activation on the p-AMPK and p-Akt protein levels. **(A,B)** Western blot analysis of the hippocampal p-AMPK **(A)** and p-Akt **(B)** protein levels at 30 min, 2 h, 1 and 5 days after GSK1016790A injection. **(C)** The decreased p-Akt protein level at 2 h after GSK1016790A injection was rescued by the AMPK antagonist CC. The decreased p-Akt protein level at 5 days after GSK1016790A injection was not affected by the AMPK agonist AICAR. ^∗∗^*p* < 0.01 vs. control mice, ##*p* < 0.01 vs. GSK1016790A, ˆˆ*p* < 0.01 vs. CC, $$*p* < 0.01 vs. AICAR.

We previously reported that acute activation of TRPV4 affected the AMPK-Akt signaling, which may be related to the TRPV4-mediated Ca^2+^ influx (Hong et al., 2016). In this study, GSK1016790A was consecutively administered for 5 days and the changes in the protein levels of p-AMPK and p-Akt at different time points after GSK1016790A injection were examined. As shown in **Figure 2**, the protein levels of p-AMPK were 172.43 ± 6.07% (*p* < 0.01), 147.69 ± 3.78% (*p* < 0.01), 53.54 ± 5.57% (*p* < 0.01), and 58.80 ± 4.89% (*p* < 0.01) of the control value at 30 min, 2 h, 1 and 5 days after the beginning of the experiment, respectively (**Figure 2A**). This result indicates that AMPK activity may enhance within 2 h after TRPV4 activation and then decrease from 1 to 5 days after TRPV4 activation. Unlike the changes in the p-AMPK protein level, the protein levels of p-Akt were 69.35 ± 5.67% (*p* < 0.01), 46.85 ± 3.47% (*p* < 0.01), 49.25 ± 5.11% (*p* < 0.01), and 50.79 ± 7.78% (*p* < 0.01) of that in the control group at 30 min, 2 h, 1 and 5 days after the beginning of the experiment, respectively (**Figure 2B**). This result indicates that Akt activity decreases upon TRPV4 activation. In order to examine whether AMPK was involved in GSK1016790A-induced inhibition of p-Akt protein level, two time points, 2 h and 5 days after the beginning of the experiment were selected, because at these two time points, AMPK activity was differently modulated by GSK1016790A. Therefore, AMPK antagonist CC and AMPK agonist AICAR was used to examine whether they could affect GSK1016790A-decreased p-Akt protein level at 2 h and 5 days after the beginning of the experiment, respectively. Notably, the decrease in the p-Akt protein level at 2 h after the beginning of the experiment was markedly attenuated by CC. The p-Akt protein level at 5 days after the beginning of the experiment was the same as that in the mice that were co-injected with GSK1016790A and AICAR (**Figure 2C**). These results indicate that upon chronic TRPV4 activation, down-regulated Akt signaling occurs most likely due to the activation of AMPK.

### Effect of TRPV4 Activation on Akt-related Signaling Pathways

**FIGURE 3:**
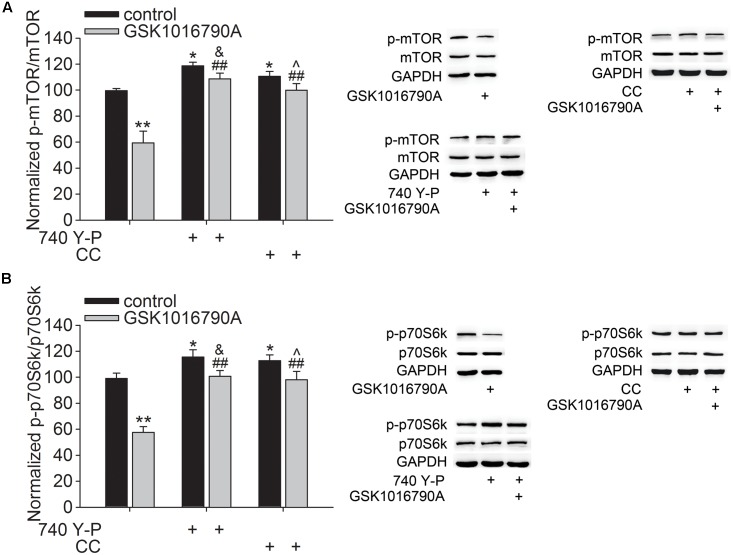
Effect of TRPV4 activation on the p-mTOR and p-p70S6k protein levels. Western blot analysis of the hippocampal p-mTOR **(A)** and p-p70S6k **(B)** protein levels in the control and GSK1016790A-injected mice. Note that the GSK1016790A-induced decreases in the p-mTOR **(A)** and p-p70S6k **(B)** protein levels were markedly attenuated by the PI3K agonist 740 Y-P or the AMPK antagonist CC. ^∗^*p* < 0.05, ^∗∗^*p* < 0.01 vs. control mice, ^##^*p* < 0.01 vs. GSK1016790A, ^&^*p* < 0.05 vs. 740 Y-P, ˆ*p* < 0.05 vs. CC.

Akt plays an important role in modulating neurite outgrowth, which is mediated through downstream signals, such as mTOR and GSK3β (Read and Gorman, 2009). In this study, the number of DCX^+^ fibers reduced after administration of GSK1016790A for 5 days. Additionally, Akt activity consistently decreased from 30 min to 5 days after the beginning of the experiment. The following experiments were performed 5 days after the beginning of the experiment to further explore the effect of TRPV4 activation on the downstream signals of Akt. Here, the protein level of p-mTOR in GSK1016790A-injected mice was 64.11 ± 3.93% of that in the control group (*p* < 0.01) (**Figure 3A**). Moreover, the phosphorylation of p70S6k (p-p70S6k), a substrate of mTOR, was also lower than that of the control value (**Figure 3B**). As shown in **Figure 3**, the decreased protein levels of p-mTOR and p-p70S6k in GSK1016790A-injected mice were markedly reversed by the administration of the PI3K agonist 740 Y-P or the AMPK antagonist CC (*p* < 0.01 in each group).

**FIGURE 4:**
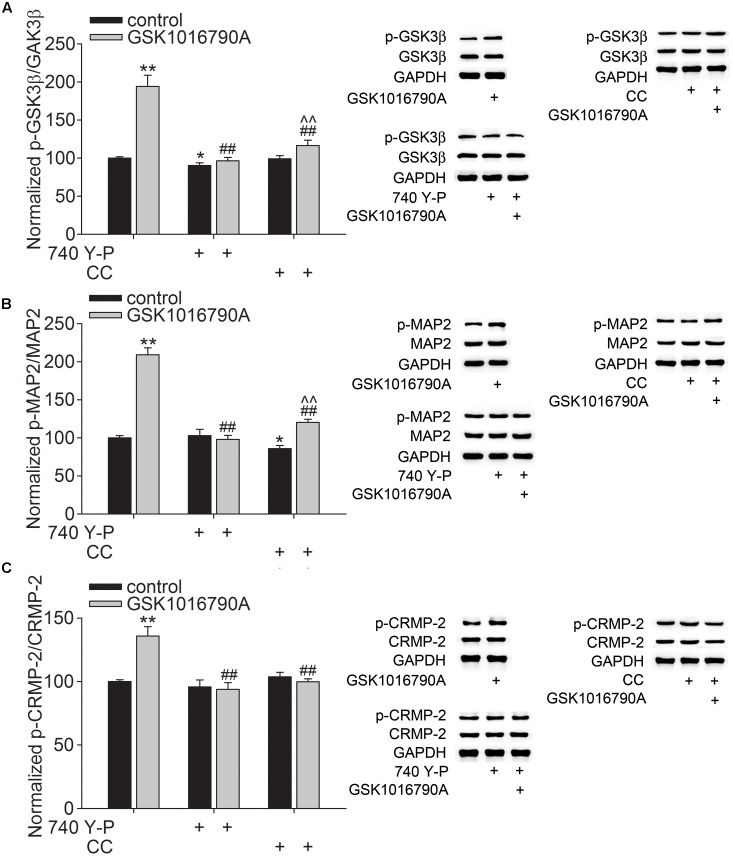
Effect of TRPV4 activation on the p-GSK3β, p-MAP2 and p-CRMP-2 protein levels. Western blot analysis of the hippocampal p-GSK3β **(A)** and p-MAP2 **(B)** and p-CRMP-2 **(C)** protein levels in the control and GSK1016790A-injected mice. Note that the GSK1016790A-induced increases in p-GSK3β **(A)** and p-MAP2 **(B)** and p-CRMP-2 **(C)** protein levels were markedly attenuated by the PI3K agonist 740 Y-P or the AMPK antagonist CC. ^∗^*p* < 0.05, ^∗∗^*p* < 0.01 vs. control mice, ##*p* < 0.01 vs. GSK1016790A, ˆˆ*p* < 0.01 vs. CC.

GSK3β is another key substrate of Akt that is critical for the specifying the axon/dendrite fate via its regulation of MAP2 and CRMP-2. As shown in **Figure 4A**, the phosphorylation of GSK3β at Y^216^ (p-GSK3β) in GSK1016790A-injected mice was markedly higher than that in the control group (*p* < 0.01). Meanwhile, the phosphorylated MAP2 (p-MAP2) and CRMP-2 (p-CRMP-2) protein levels in GSK1016790A-injected mice were markedly higher than the control values (*p* < 0.01 in each group) (**Figures 4B,C**). Here, the GSK1016790A-induced increase in the p-GSK3β, p-MAP2 and p-CRMP-2 protein levels were markedly attenuated by 740 Y-P or CC (**Figure 4**).

### Involvement of the AMPK and Akt Signaling Pathways in the TRPV4 Activation-Induced Inhibition of the Dendritic Arborization of Newborn Neurons in the Hippocampal DG

**FIGURE 5:**
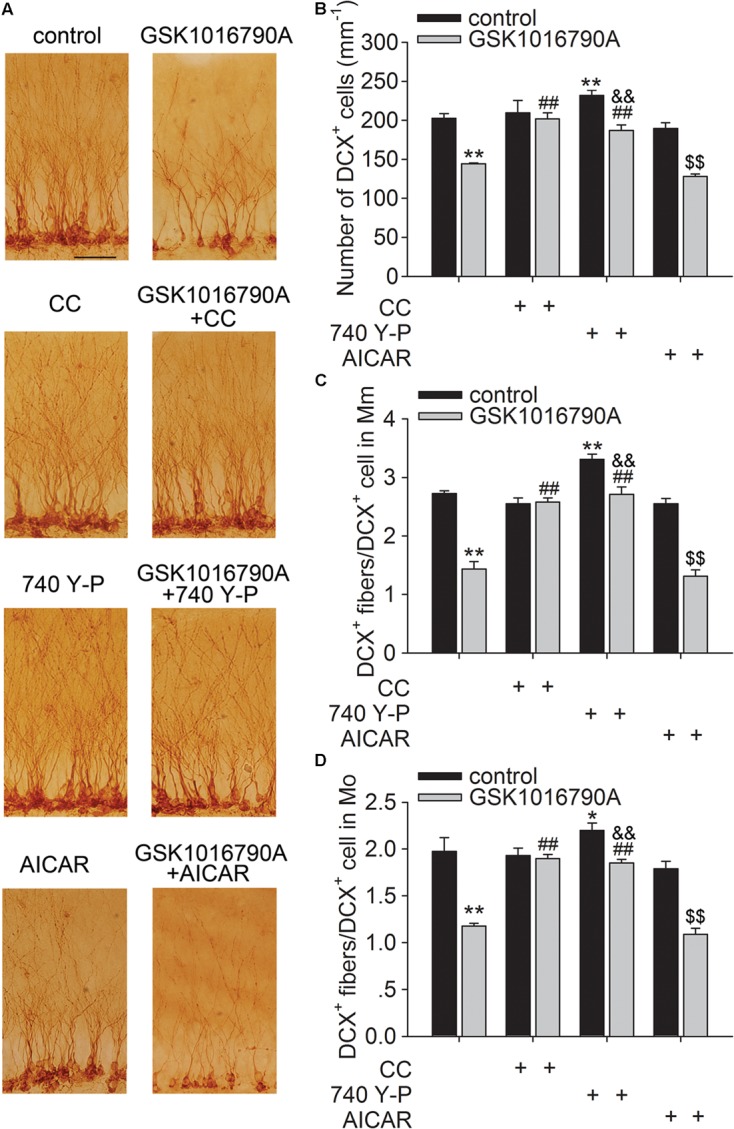
Involvement of the AMPK and Akt signaling in the TRPV4 activation-induced impairment of dendritic arborization. DCX immunostaining **(A)** and the bar graph show that GSK1016790A-induced decreases in DCX^+^ cells **(B)** and DCX^+^ fibers in the Mm **(C)** and Mo **(D)** subregions were attenuated by CC (AMPK antagonist) and 740 Y-P (PI3K agonist), but were unaffected by AICAR (AMPK agonist). ^∗^*p* < 0.05, ^∗∗^*p* < 0.01 vs. control mice, ##*p* < 0.01 vs. GSK1016790A, &&*p* < 0.01 vs. 740 Y-P, $$*p* < 0.01 vs. AICAR.

As shown in **Figure 5**, the number of DCX^+^ cells in the SGZ was markedly higher in mice co-injected with GSK1016790A and CC or 740 Y-P than in mice injected with GSK1016790A alone (*p* < 0.01 in each group). DCX^+^ fibers in Mm and Mo increased markedly in the mice that were co-injected with GSK1016790A and CC or 740 Y-P, and the values were markedly different from those in mice that were injected with GSK1016790A alone (*p* < 0.01 in each group). By contrast, the numbers of DCX^+^ cells and DCX^+^ fibers in the Mm and Mo subregions in GSK1016790A-injected mice were nearly the same as those in the mice that were co-injected with GSK1016790A and AICAR. Our data suggest that the activation of AMPK and inhibition of the Akt signaling pathway are likely involved in the TRPV4-induced decrease of newborn neurons and the impairment of dendritic arborization of newly generated neurons.

## Discussion

Neurite growth is a critical process of neurogenesis. Newborn neurons extend axonal and dendritic projections and establish new synaptic connections to the existing hippocampal circuitry (Alvarez-Buylla and Garcia-Verdugo, 2002; Zhao et al., 2006). Ca^2+^ has been proven to be an important regulator of neurite growth. Axon outgrowth and dendritic development occur within the optimal [Ca^2+^]_i_ level, and this process may be inhibited when the [Ca^2+^]_i_ level is below or above this optimal [Ca^2+^]_i_ level (Mattson, 1987; Redmond and Ghosh, 2005; Zheng, 2007). As Ca^2+^-permeable channels, some TRP family members induce Ca^2+^ influx, leading to increases in [Ca^2+^]_i_. Accordingly, these channels have been shown to play a role in modulating neurite growth (Benemei et al., 2015). For example, TRPC5 participates in controling hippocampal neurite length and growth cone morphology, and activation of TRPC5 inhibits neurite extension as well as a rapid vesicular trafficking mechanism regulated by growth factors (Greka et al., 2003; Hui et al., 2006). Inhibition of TRPM2 markedly increases axonal growth, whereas over-expression of it inhibits axonal growth (Jang et al., 2014). Blockage of TRPV1 promotes neurite outgrowth after sciatic nerve injury (Ren et al., 2015). However, activation of TRPV2 leads to axon outgrowth in the developing dorsal root ganglion and motor neurons (Cohen et al., 2015). A gain-of-function of TRPV4 mutation increased its Ca^2+^ channel activity, which underlies the pathogenesis of TRPV4-linked axonal neuropathies (Fecto et al., 2011). Activation of TRPV4 may increase the [Ca^2+^]_i_ in hippocampal neurons (Shibasaki et al., 2007). In this study, DCX^+^ fibers was expressed as DCX^+^ fibers/DCX^+^cell and DCX^+^ fibers in Mm and Mo subregions were reduced by TRPV4 agonist (**Figure 1**), suggesting that the density and length of dendrite fibers per newborn neuron were impaired by TRPV4 activation. We previously reported that application of GSK1016790A did not affect the number of DCX^+^ fibers (Tian et al., 2016). In our previous study, GSK1016790A was consecutively injected for 5 days and DCX staining was performed 14 days after the last GSK1016790A injection (Tian et al., 2016). In this case, GSK1016790A was supposed to be absent 7 days after injection and the result that GSK1016790A had no effect on DCX^+^ fibers may not truly reflect the direct effect of TRPV4 activation on the dendritic arborization of newborn neurons. In this study, DCX staining was performed on the same day of the last GSK1016790A injection, and therefore, this inconsistency about the effect of TRPV4 activation on the DCX^+^ fibers is likely due to the difference in drug application protocols between the present and previous studies (Tian et al., 2016).

Akt is a key mediator of several aspects of neurite outgrowth, including elongation, branching and caliber (Read and Gorman, 2009). Previous reports from our group showed that activation of TRPV4 reduced the phosphorylation of Akt (Jie et al., 2015, 2016; Hong et al., 2016). AMPK is a serine/threonine kinase that functions as an important sensor in energy homeostasis in mammalian cells. In addition to an increased ratio of intracellular AMP to ATP, Ca^2+^/calmodulin-dependent protein kinase kinase-β can also activate AMPK in response to an increased [Ca^2+^]_i_ (Hawley et al., 2005). We recently reported that acute application of a TRPV4 agonist caused an increase in the phosphorylation of AMPK, which was related to the TRPV4-mediated Ca^2+^ influx (Hong et al., 2016). AMPK has been shown to inhibit neuronal development at multiple stages, during both axon outgrowth and dendrite growth and arborization (Ramamurthy et al., 2014). Activation of AMPK pathway can suppress axon initiation and neuronal polarization through interfering with PI3K signaling (Amato et al., 2011; Amato and Man, 2012). Application of AMPK antagonist CC promotes the dopaminergic dendrite outgrowth (Wakita et al., 2014). These reports prompted us to study whether the AMPK and Akt signaling pathways were involved in the TRPV4-induced inhibition of the dendritic arborization of newborn neurons. Here, we first examined the phosphorylation of AMPK and Akt at different time points after GSK1016790A treatment. The present results showed that the AMPK activity increased within 2 h and then decreased from 1 to 5 days after the beginning of the experiment, whereas the Akt activity consistently decreased from 30 min to 5 days after the beginning of the experiment. Notably, the decrease in the p-Akt protein level in GSK1016790A-injected mice was markedly blocked by the AMPK antagonist CC, but it was unaffected by the AMPK activator AICAR (**Figure 2**). Therefore, during chronic TRPV4 activation, the initial activation of AMPK is probably responsible for the inhibition of Akt signaling. We also found that the GSK1016790A-induced decrease of DCX^+^ cells and DCX^+^ fibers was markedly blocked by the PI3K agonist 740 Y-P or by AMPK antagonist CC. By contrast, the application of AMPK agonist AICAR did not have a similar effect in GSK1016790A-injected mice (**Figure 5**). Akt signaling also plays an important role in neuronal survival and previous studies show that activation of TRPV4 results in neuronal death, which is mediated, at least partially, by inhibiting Akt signaling (Luo et al., 2003; Jie et al., 2015, 2016). Therefore, our findings suggest that AMPK activation and the related down-regulation of Akt signaling are involved in the TRPV4-induced decrease of newborn neurons and the dendritic arborization of single neuron. Nevertheless, it was still unclear how AMPK activity was subsequently inhibited when TRPV4 was chronically activated. A previous study showed that activation of extracellular signal-regulated kinase 1/2 (ERK1/2) is responsible for dephosphorylation of AMPK at Thr^172^ caused by α- melanocyte-stimulating hormone (Damm et al., 2012). Of note, activation of TRPV4 has been proven to increase ERK1/2 activity (Jie et al., 2015; Tian et al., 2016). More experiments are needed to clarify whether the enhanced ERK1/2 is involved in the decrease in AMPK activity during the later stage of TRPV4 activation. The present data showed that the TRPV4-induced decrease in the p-Akt protein level was not affected by AICAR, indicating that other factors contribute to the inhibition of Akt signaling during chronic TRPV4 activation.

Mammalian target of rapamycin and GSK3β, two major substrates that are downstream of Akt, have been identified to play key roles in Akt-mediated neurite growth (Read and Gorman, 2009). Activation of the PI3K-Akt-mTOR signaling pathway can phosphorylate p70S6k, which plays a fundamental role in axonal and dendritic growth. In cultured hippocampal neurons, the dendritic complexity was reduced by inhibition of PI3K and knockdown of mTOR or p70S6k (Jaworski et al., 2005). In AD model mice, the neurite growth of newborn neurons in the hippocampal DG is impaired with reduced phosphorylation of Akt and mTOR (Li L. et al., 2010). Here, decreased p-mTOR and p-p70S6k protein levels were found in GSK1016790A-injected mice, and this inhibitory effect caused by TRPV4 activation was markedly blocked by 740 Y-P or CC (**Figure 3**). Together with the above discussion, these results indicate that activation of TRPV4 may increase AMPK activity and then lead to inhibition of PI3K-Akt-mTOR-p70S6k signaling pathway. GSK3β has been proven to play an important role in neuronal morphogenesis. GSK3β activity is inversely correlated with PI3K-Akt signaling activity (Cross et al., 1995). One of the downstream targets of GSK3β is MAP2, which is important for microtubule polymerization and dendrite elongation (Sánchez et al., 2000; Lim and Walikonis, 2008). Phosphorylation of MAP2 causes it to lose its ability to effectively associate with the microtubules. Akt activation was shown to inactivate GSK3β and consequently reduce phosphorylation of MAP2, which can promote microtubule polymerization and dendrite elongation (Lim and Walikonis, 2008). Another important downstream target of GSK3β is CRMP-2 that is critical for promoting axon specification and neurite extension through reorganization of actin filaments, regulation of microtubule assembly, endocytosis of adhesion molecules and also axonal protein trafficking (Yoshimura et al., 2005, 2006). GSK3β phosphorylates CRMP-2 to decreases its tubulin heterodimer binding ability (Yoshimura et al., 2005, 2006). In the present study, the p-GSK3β (Y^216^) protein level increased significantly in GSK1016790A-injected mice, indicating higher activity of GSK3β upon TRPV4 activation (Bhat et al., 2000). In addition to the increased p-GSK3β protein level, the p-MAP2 and p-CRMP-2 protein levels were increased by GSK1016790A treatment, indicating a decrease in the MAP2 and CRMP-2 function upon TRPV4 activation (**Figure 4**). Here, the increase in the p-GSK3β (Y^216^), p-MAP2 and p-CRMP-2 protein levels was markedly lower in the mice that were co-injected with GSK1016790A and 740 Y-P or and CC (**Figure 4**); this finding indicates that activation of TRPV4 may increase AMPK activity to down-regulate PI3K-Akt signaling, then increase GSK3β activity, and ultimately decrease MAP2 and CRMP-2 function.

**FIGURE 6:**
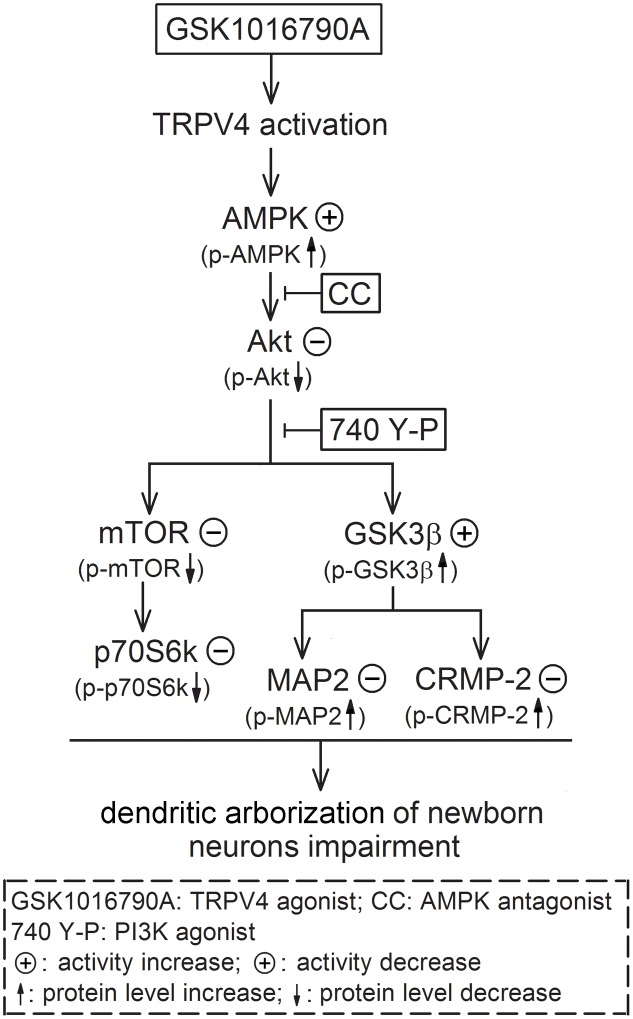
Possible mechanisms underlying TRPV4 activation-induced impairment of dendritic arborization of newborn neurons. Activation of TRPV4 impairs the dendritic arborization of newborn neurons in the adult hippocampl DG and this effect is dependent on increasing AMPK activity and inhibiting Akt. The inhibited Akt leads to down-regulate mTOR-p70S6k signaling and increase of GSK3β activity; the increased GSK3β activity results in inhibition of MAP2 and CRMP-2 function ultimately.

The present results suggest that activation of AMPK and inhibition of Akt signaling are likely responsible for TRPV4-induced impairment of dendritic arborization of newborn neurons, which is mediated by downstream substrates of Akt, including mTOR and GSK3β (**Figure 6**). In this study, we did not provide the direct evidence for TRPV4 activation-induced modulation of axon outgrowth. Concerning the important role of AMPK, Akt and the related substrates in the modulation of axonogenesis, the activation of AMPK and the down-regulation of Akt signaling provide a possibility that the axon outgrowth of newborn neurons may be impaired by TRPV4 activation. More experiments are needed to prove this speculation. Here it is noted that besides the decrease of DCX^+^ fibers, the number of DCX^+^ cells was also reduced by activation of TRPV4. It has been reported that apoptosis of newborn cells occurs during adult neurogenesis (Sierra et al., 2010). In our previous study, the inhibited Akt signaling is involved in TRPV4 activation-induced apoptosis and therefore this action may be a reason for the present decreased DCX^+^ cells (Jie et al., 2015). Neurite growth is important for the survival of newborn neurons and impaired neurite growth may result in a decline of newborn neurons in the pathological condition (Li L. et al., 2010). In the present study, we did not examine the dying process of DCX^+^ cells upon TRPV4 activation, but the present reduced DCX^+^ fibers indicates that these newborn neurons were already “unhealthy” cells and their subsequent survival might be impaired. Collectively, TRPV4 activation-induced impairment of dendrite morphology may result from the decreased newborn neurons and the impairment of dendritic arborization of single neuron. As a Ca^2+^-permeable channel, TRPV4 may be a new potential target for modulating neurite growth. The present study also provides more evidence that TRPV4 activation probably plays an important role in the modulation of several processes of adult hippocampal neurogenesis.

## Author Contributions

YT, MQ, ZW, and SS performed experiments; CW, ZS, and YL analyzed data; LeC designed experiments and wrote the article; YD and LiC revised the manuscript and all authors approved the final version.

## Conflict of Interest Statement

The authors declare that the research was conducted in the absence of any commercial or financial relationships that could be construed as a potential conflict of interest.
